# *Cordyceps militaris* Reduces Oxidative Stress and Regulates Immune T Cells to Inhibit Metastatic Melanoma Invasion

**DOI:** 10.3390/antiox11081502

**Published:** 2022-07-30

**Authors:** Yuan-Hong Lan, Yun-Sheng Lu, Ju-Yu Wu, Hsu-Tung Lee, Penjit Srinophakun, Gizem Naz Canko, Chien-Chih Chiu, Hui-Min David Wang

**Affiliations:** 1 Department of Medical Laboratory Science and Biotechnology, ASIA University, Taichung 413, Taiwan; brianblue98@gmail.com; 2 Taiwan Agriculture Research Institute, Council of Agriculture, Taichung 413, Taiwan; yunsheng@tari.gov.tw; 3 Program in Tissue Engineering and Regenerative Medicine, National Chung Hsing University, Taichung 402, Taiwan; xytmilk@gmail.com; 4 The Department of Neurological Institute, Taichung Veterans General Hospital, Taichung 402, Taiwan; sdlee@vghtc.gov.tw; 5 Chemical Engineering Department, Faculty of Engineering, Kasetsart University, 50 Ngamwongwan Rd., Ladyao, Jatujak, Bangkok 10900, Thailand; fengpjs@ku.ac.th; 6 Graduate Institute of Biomedical Engineering, National Chung Hsing University, Taichung 402, Taiwan; gizemcanko@gmail.com; 7 Department of Biotechnology, Kaohsiung Medical University, Kaohsiung 807, Taiwan; cchiu@kmu.edu.tw; 8 Graduate Institute of Medicine, College of Medicine, Kaohsiung Medical University, Kaohsiung 807, Taiwan; 9 Department of Medical Laboratory Science and Biotechnology, China Medical University, Taichung 404, Taiwan

**Keywords:** *Cordyceps militaris*, B16F10, lung metastatic melanoma, tumor microenvironment, T cell

## Abstract

In this study, the water extract of *Cordyceps militaris* (Linn.) Link (CM) was used as a functional material to investigate the inhibitory mechanisms on B16F10 and lung metastatic melanoma (LMM) cells. Reducing power, chelating ability, and 2,2-diphenyl-2-picrylhydrazyl (DPPH) assays were applied for antioxidative capacities, and we obtained positive results from the proper concentrations of CM. To examine the ability of CM in melanoma proliferation inhibition and to substantiate the previous outcomes, three cellular experiments were performed via (3-(4,5-dimethylthiazol-2-yl)-2,5-diphenyltetrazolium bromide, MTT, a tetrazole) assay, cell migration, and invasion evaluation. The addition of CM to the incubation medium increased the number of CD8+ T cells significantly, which improved the immunogenicity. This study showed that CM exhibits various biological capabilities, including antioxidation, anti-tumor, tumor invasion suppression, and T cytotoxic cell activity promotion.

## 1. Introduction

Many studies show that the reactive oxygen species (ROS) in the tumor microenvironment is a double-edged sword for cancer curing. Different ROS levels lead to different outcomes to various aspects of cancer. When the tumor microenvironment has a higher ROS level, it could induce inflammatory responses and prolonged ROS production could lead to chronic inflammation. It can also cause a series of reactions to trigger tumor progression and metastasis [1]. Moreover, the amount of oxidative stress increase can also elevate the resistance of cancer cells to drugs [2]. Still, the excessive rise of oxidative stress could damage cells to death directly by promoting apoptosis. In contrast, the cancer cell could also activate the apoptosis process when the tumor microenvironment has a mild ROS level, which is lower than the minimum requirement for the cellular response. Some studies confirm that a sharp ROS level decrease could significantly inhibit cell proliferation [3,4].

Melanoma is an aggressive tumor that quickly metastasizes and strongly resists chemotherapy drugs. This characteristic has caused the survival rate of patients to remain low in recent years [5] Even though the current medical treatments are not effective enough to treat metastatic melanoma, stimulation of the cancer cells’ death is an effective strategy for cancer chemotherapy. The process of causing cell death is named programmed cell death, which has two forms: autophagy and apoptosis. The main differences between autophagy and apoptosis are the cell morphology and the metabolic pathway. Autophagy is an evolutionary self-preservation mechanism through the dysfunctional cells removing and recycling parts of them toward cellular repair [6]. Apoptosis changes in cell morphological characteristics: cell contraction, roundness, and blistering of cell nuclei, condensation, marginalization of chromatin, and undetectable transformations of cellular organelles, and those changes cause cell death [7].

The water extract of *Cordyceps militaris* (Linn.) Link (CM, orange caterpillar fungus or Yong Chong Cao in Chinese) is a medicinal fungus that belongs to the family of Cordycipitaceae. Various insects as a host can be infected by CM in the field, including lepidopteran larvae and pupae. In addition, CM can be artificially cultivated on a rice medium to harvest the sporocarp. Because the medicinal properties of CM are like those of *Cordyceps sinensis* (CS), it is used widely as an alternative to CS in health-promoting supplements. *Cordyceps sinensis* (CS) is a fungal parasite from lepidopteran larvae. The fungus attacks the caterpillar in late autumn and devours the host leisurely. By early summer, the fungus-infected caterpillar is dead, and the fruiting body sticks out of its head. Moreover, its particular life cycle is called Cordyceps Sinensis in Chinese [8]. Furthermore, recent reports have revealed that both CM and CS have anti-inflammatory and antioxidative effects. Cordyceps has a significant impact. Cordycepin, as a biologically active compound of *Cordyceps militaris*, is very similar in structure to cellular nucleosides and adenosine and contains a purine (adenosine) nucleoside molecule linked to a ribose moiety, which acts as a nucleoside analogue [9,10]. In addition, also because cordycepin alone is widely explored for its anticancer/antioxidant activity, it has strong pharmacological and therapeutic potential to cure many dreaded diseases in the future, such as respiratory [11], renal [12], hepatic, and nervous system diseases [13]. Therefore, we assume a couple of combinations in CM could provide antioxidation functions [14]. Many valuable chemicals in natural products could interact to show anti-cancer ability, activate the immune system, and promote the progress of apoptosis [2,15]. Moreover, the interactions between the chemicals in natural products are not precise yet, and it is challenging to figure out the process.

T cells play an essential role in the immune system because they have an exact and circumspect mechanism to adjust the programmed cell death of cancer cells. Therefore, T cells can ensure no harm to the host during the process at any stage during the apoptosis [16]. Furthermore, some studies confirm that activating T cells could be an excellent strategy to cure malignant tumors, especially melanoma because a robust immune response could be mobilized by melanoma generally [12,16]. Therefore, this study will investigate the function of CM in antioxidant, anti-melanoma, and stimulating the capability of CD8+ T cells.

## 2. Materials and Methods

### 2.1. Reagents

1,1-Diphenyl-2-picrylhydrazyl, dimethyl sulfoxide (DMSO), potassium ferricyanide, trichloroacetic acid, ethylene diamine tetra-acetic acid, horseradish-peroxidase, ${\mathrm{FeCl}}_{3},{\mathrm{FeCl}}_{2}\cdot 4H_{2}O$, fetal bovine serum (FBS), and Vitamin C were obtained from GIBCO BRL (Gaithersburg, MD). 3-(4,5-dimethylthiazol-2-yl)-2,5-diphenyltetrazolium bromide (MTT), and 5(6)-carboxy-2′,7′-dichlorofluorescein diacetate (DCFDA) were purchased from Sigma Chemical.

### 2.2. Cell Lines and Cell Culture Materials

B16F10 melanoma cells were purchased from the Bioresource Collection and Research Center (BCRC, Hsinchu, Taiwan). The generation of LMM is based on the injection of B16F10 from the tail vein of mice. After waiting for 3–4 weeks to establish the tumor, it can extract the cancer from the lung and culture, which is LMM after subculture. B16F10 and LMM were grown in Dulbecco’s Modified Eagle’s Medium (DMEM, Sigma-Aldrich Corp., Burlington Township, NJ, USA) supplemented with 10% heat-inactivated fetal bovine serum (FBS, Sigma-Aldrich Corp. Burlington Township, NJ, USA), and 1% antibiotic-antimycotic (100X) (Thermo Fisher Scientific Inc., Waltham, MA, USA) and maintained at 37 °C in a 5% CO_2_ humidified incubator.

### 2.3. CM Extraction Process and Preparation

The CM was taken from the Mushroom Laboratory of the Taiwan Agricultural Research Institute (TARI), Executive Yuan. *Cordyceps militaris* (Linn.) Link (strain number: CM03) was kindly provided by Dr. Jin-Torng Peng and Yuegi Co. (Taipei, Taiwan). CM was authenticated by Dr. Yun-Sheng Lu later cultivated under controlled temperature (23 ± 4 °C) and humidity (80 ± 5%) at the Mushroom Laboratory of TARI, Taichung, Taiwan.

**Figure 1:**
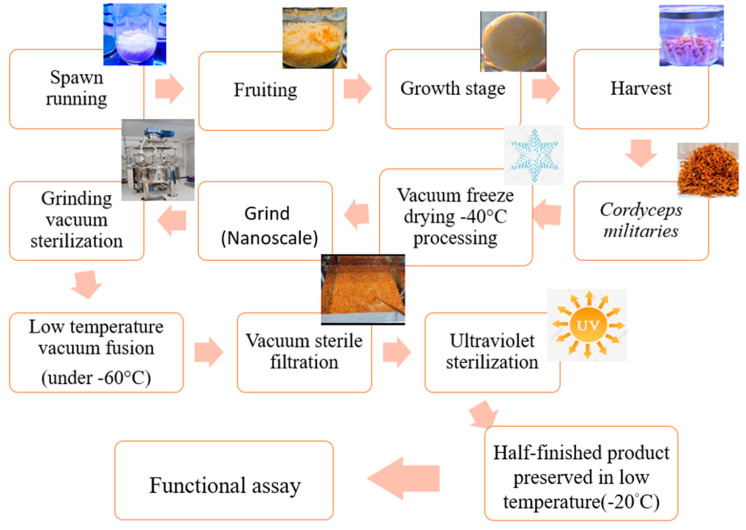
The extraction process using freeze-drying technology.

Water extraction of CM was carried out by the collaborator Qiqi Industrial Co., Ltd. The resulting product was freeze-dried at −40 °C and then pulverized with super micron. After the vacuum sterilization, the vacuum decompression fusion was performed in an environment below −60 °C, vacuum filtration and ultraviolet sterilization were used, and the semi-finished products were finally stored at low temperature. The water was partially concentrated and freeze-dried to obtain a stock solution (200 mg/mL). The diluted solution was then used for the DMEM dilution (Figure 1).

### 2.4. Reducing Power Assay

This system was used to quantify the reduction ability of antioxidants. First, 100 μL hydroxy anisole (BHA) was chosen to be a positive control. Then, 2.5 μL of the sample solutions of different concentrations (1–1000 μg/mL) mixed with 85 μL potassium and 2.5 μL potassium ferricyanide (20%, *w/v*) and phosphate buffer (0.2 M, pH 6.6), then let stand at 50 °C for 20 min. The reaction was terminated by adding 160 μL of trichloroacetic acid solution (10%, *w/v*) to the extractant. Later, 75 μL from the supernatant was removed and added into a 96-well plate, then 25 μL of ferric chloride (0.2%, *w/v*) was added, and the mixture was placed in the dark for 30 min. Finally, the absorbance value was measured at 700 nm. Results showed that when the absorbance level of the reaction mixture increases, the reducing power of the sample gets stronger, and they are directly proportional [17].

### 2.5. Ferrous Ion Chelating Assay

The antioxidant properties of iron were analyzed by the results of iron chelation; however, removal of iron ions is one of the things that can be achieved. In the medium of chelating agent, the structure cause complex was destroyed, which caused the complex ferrous ion to change from crimson restoration. The determination was based on the color reaction of the complex of ferrous ion and ferrozine at 562 nm. The lower the absorbance, the better the metal chelating activity. Then, 1 μL of CM of different concentrations (1–1000 μg/mL) was taken and mixed with 10 μL of ${\mathrm{FeCl}}_{2}$ (2 mM), let stand for 5 min, then 69 μL of methanol and 20 μL (5 mM) of ferrozine were added. The sample was vigorously shaken to start the reaction, later the mixture was kept at 25 °C for 10 min. The absorbance value of the mixture was measured at 562 nm. EDTA was used as a positive standard [15]. The calculation formula was:

$$\mathrm{Scavenging}\;\mathrm{activity}\;(\%)\;=\frac{\left(A_{\mathrm{control}}-A_{\mathrm{sample}}\right)}{A_{\mathrm{control}}}\times 100\%$$

where *A*_control_ = the absorbance of the control and *A*_sample_ = the absorbance of the sample.

### 2.6. DPPH Radical Scavenging Activity Assay

The antioxidant activity of the samples was measured by using DPPH. A DPPH solution (60 mM; 99 μL) in methanol was added to each compound’s solution (1 μL). Vitamin C at 100 μg was set as a positive control. After 30 min, we measured the absorption at optical density (OD) of 517 nm [4]. Free radical scavenging capacity (percentage) was calculated according to the given formula above.

### 2.7. Cell Proliferation Assay by MTT Assay

The method used for the evaluation of the cell viability, proliferation, and cytotoxicity was MTT assay. This method was based on the succinate dehydrogenase of the mitochondria of living cells to reduce MTT to purple crystalline formazan (insoluble in water) and deposit it in the cells, while dead cells do not react the same way as threatening cells. First, in a 96-well plate, the cells were incubated at 1 × 10^4^ cells/well for 24 h. After incubation, the medium was removed then replaced with fresh medium and *Cordyceps* water extract (1, 5, 25, 125, 500, and 1000 μg/well). After one more day of incubation, the supernatant was removed, and 100 μL from 0.5 μg/mL MTT/DMEM solution was added to each well and placed in the incubator for 2 h. Carefully the solution was removed, 100 μL DMSO was added into the wells to dissolve the crystals, the well plate was placed on the shaker plate, and the plate was gently shaken for 10 min in the dark. The absorbance value was measured at 570 nm (BioTek, Shoreline, WA, USA) [18].

### 2.8. Detect Reactive Oxygen Species by DCFDA Stain

To detect that intracellular PMA upregulates the level of intracellular oxidative stress, we used 2’,7’-dichlorofluorescein diacetate (DCFDA, Sigma-Aldrich Chemical Corp., D6883); The DCFDA stock solution was prepared by mixing it with DMSO, then diluted into a working solution (20 μMol) containing PBS. The cells were cultured in a 6-well plate at a density of 1 × 10^5^ for 1 day, later treated with three different concentrations (5, 25, and 125 μg/mL) of water extraction of CM. The harvested cells were mixed with the working solution at 37 °C for 30 min and analyzed with a flow cytometer at 488 nm laser (Guava^®^ easy yet 5HT, Merck KGaA, Darmstadt, Germany). The fluorescence intensity of the cells should be detectable at 530 nm [12].

### 2.9. Inhibiting Effects on Melanoma Cell Migration by Wound Healing Test

The cell migration experiment was completed mainly to detect the inhibitory effect of the threatening analyte through the wound healing test, with minor modifications. In short, 5 × 10^5^ cells were seeded in a 6-well plate and incubated until grown to complete confluence. Then, a 200 μL plastic pipette tip was used to form a clear wound. The area was washed with PBS and then treated with the prepared sample. The cell crawling was checked after 24 h of wound formation. Cell migration and movement through the wound area were calculated by using the free software ImageJ [18].

### 2.10. Boyden Invasion Assay

The Transwell Invasion Test was used to study the influence of the analyte on the invasion of melanoma cells. It was necessary to inoculate the top of Boyden’s Chamber with a serum-free medium to be able to nurture B16F10 and LMM cells. The 8-micron holes in the upper insert (ThinCert™, Greiner, Frickenhausen, Germany) were pre-coated with the base matrix which was Matrigel. The cells were stored in three different concentrations (5, 25, and 125 μg/mL) of CM and a solvent containing 10% FBS, which was regarded as a control cell. Cells on the lower surface of the filter membrane needed to be fixed by using paraformaldehyde and Giemsa staining counted to analyze the results [4].

### 2.11. Quantitative Real-Time Reverse Transcription Polymerase Chain Reaction (qRT-PCR) Analysis

The fluorescent signal was generated by a unique probe formed as qRT-PCR, which is a detection method using the StepOnePlus™ system (Thermo Fisher Scientific Inc., USA) fluorescence detection technology to sense each cycle. It detects and records the fluorescence intensity of each cycle and calculates real-time quantitative data. For qRT-PCR, the reaction mixture uses SYBR Green Master Mix (Qiagen, Valencia, CA, USA) using primers and templates. We used TRIzol (Invitrogen, Waltham, MA, USA) to extract RNA from B16F10 and LMM cells and then used a reverse transcription kit (Takara, Shiga, Japan) to generate DNA. For qRT-PCR using primers, single-stranded DNA was formed from the sample, then the primers were combined for dsDNA formation, and then SYBR Green dsDNA (Roche, Basel, Switzerland) was added. All steps were repeated for 40 amplification cycles, and the resultant was passed through the fluorescence detection system [18].

### 2.12. Western Blotting

In this step, 1 × 10^6^ B16F10 cells and lung metastasis cells were seeded in 12 wells and treated for 24 h. After treatment with *Cordyceps* overnight, the cells were collected and lysed with lysis buffer (Thermo Scientific Pierce RIPA buffer) for cellular protein extraction. The lysate was centrifuged at 1200 rpm for 15 min, and the protein concentration was measured using the bicinchoninic acid (BCA) protein assay kit (Pierce, Rockford, IL, USA). Sodium dodecyl sulfate-polyacrylamide gel electrophoresis (SDS-PAGE) was used for the protein separation then the proteins were electro-transferred on the gel to a polyvinylidene fluoride (PVDF) membrane (Pall Life Science, Ann Arbor, MI, USA). The membrane was blocked with blocking buffer (Pierce TOOL Speed PLUS blocking reagent) and washed three times with TBST containing Tween-20 (Tris-buffered saline, pH 8.0) for 5 min each. The membrane and the corresponding primary antibody were left on a shaker overnight at 4 °C, later washed 3 times, blocked again, and the corresponding primary and secondary antibodies were shaken at room temperature for 60 min. West Femto Maximum Sensitivity Substrate Kit (SuperSignal, Rockford, IL, USA) was used to visually detect the signal through enhanced chemiluminescence (ECL) detection [12].

### 2.13. Statistical Analysis

All experiments were performed in triplicate in each assay and expressed as mean ± standard error. The difference between the control and *Cordyceps*-treated cells in the in vitro assay was analyzed by Student’s *t*-test because *p* value < 0.05 was considered statistically significant [12].

## 3. Results

### 3.1. Antioxidant Activities in CM

In this study, the antioxidative capabilities of CM were evaluated by reducing power, ferrous ion chelating activity, and DPPH scavenging ability, which was used frequently as detection for the eradication of free radical quantities by observing the color-change. One of the CM bio functions was assumed as a powerful antioxidant, which played an essential role in absorbing and neutralizing free radicals. The cell composition, such as lipid and protein, would be oxidated because of the excessive construction and accumulation of free radicals. There are different mechanisms to eliminate ROS: quenching singlet, triplet oxygen, or decomposing peroxides, and those three assays could distinguish cases. Table 1 confirmed that CM had dose-dependent manner competencies in the three experiments above, especially reducing power.

The reducing power assay is a standard method for detecting the ability to lose electronics in the materials by altering color from yellow to green. The level of color diversity depended upon the capability of the examined antioxidants. The Fe^3+^/ferricyanide complex could be reduced into a ferrous form by the presence of a suitable substance. The reducing power of various concentrations of CM is demonstrated in Table 1, which showed a strong potential of reduction compared to the positive group. The consequence revealed a linear bio-functional response in different suitable CM concentrations. For example, at 500 μg/mL CM, the antioxidant level exhibited higher than 50% of the antioxidant properties. The experiment demonstrated CM provided a strong potential in ROS elimination.

The ferrous ion chelating assay could examine the compounds as chelating agents to disrupt the ROS construction by lightening the red complex. In addition, different concentrations of CM would compare to the metal removal capability of EDTA, which is the positive control. The ferrous ion chelating ability of CM presented a concentration-dependent trend. At 125 μg/mL CM, the chelating power of metal exceeded 50%, which was precisely 52.9% compared to the EDTA group. The chelating level rose slightly when the consistency of CM escalated, and it was about 77.7% at 1000 μg/mL. However, the lower concentration of CM could not expose enough antioxidative capacity. At the concentration of 5 μg/mL, CM had a minor level (26.6%) compared to the positive control.

DPPH free radical scavenging system is a proven assay for evaluating the substance’s capability as a hydrogen donor to remove the stable radical DPPH and turn it into diphenyl-picrylhydrazine. Therefore, the assay color will shift from deep blue to light yellow when the element can absorb the free radicals. CM showed a limited antioxidant capacity, which directly correlated with the CM dosages. The free radical emission capability was under 50% when 125 μg/mL CM was conducted, only 40.7% and 1000 μg/mL CM was 69.7%; nevertheless, the antioxidative ability was proportional to the concentration of CM.

ROS promoted cancer cells to secrete various inflammatory factors, which could strengthen the metastasis capability of cancer cells, increase the proliferation of vascular endothelium, and inhibit the immune system. Furthermore, ROS could create the immunosuppression microenvironment of tumors because of the continuous oxidant generation. As a result, cancer cells could continually develop and deteriorate. Therefore, the bio function of alleviating chronic inflammation and preventing cancer was related to the existence of antioxidants. The experiments above confirmed the adequate ability to eliminate ROS in CM, which was more effective in reducing power. The ferrous ion chelating and DPPH assay likewise had a mild ability in reducing oxidant.

### 3.2. Effects of CM on the Proliferative Viability of B16F10 and LMM Cells

**Figure 2:**
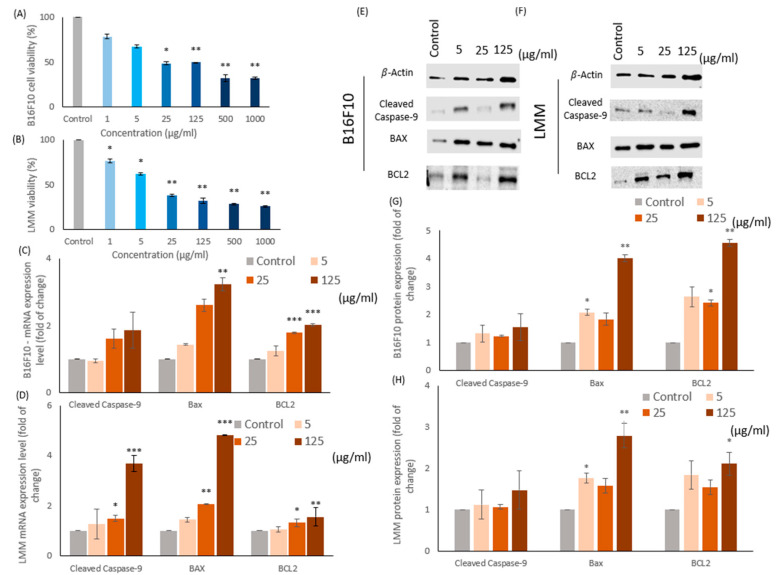
The cell viability of (**A**) B16F10 and (**B**) LMM was examined by MTT assay in different concentrations of CM. The mRNA levels of cleaved caspase-9, Bax, and BCL2 in (**C**) B16F10 and (**D**) LMM were tested after incubation in 5, 25, and 125 μg/mL CM. Protein levels of cleaved caspase-9, Bax, and BCL2 in (**E**) B16F10 and (**F**) LMM were detected by Western blotting after 5, 25, and 125 μg/mL CM were conducted. The protein quantity of cleaved caspase-9, Bax, and BCL2 in (**G**) B16F10 and (**H**) LMM were analyzed by ImageJ. *p < 0.05, ** *p* < 0.01, *** *p* < 0.005.

In this study, two melanoma-related cell lines, B16F10 and lung metastatic melanoma (LMM) cells, were incubated 24 h in 1, 5, 25, 125, 500, and 1000 μg/mL of CM independently. The cell viabilities were tested by MTT assay, which could monitor the cell proliferative viability in observing the color of chemical alternated into purple. Figure 2A,B illustrated the results of the MTT assay for B16F10 and LMM separately in different CM concentrations. In general, the increase of CM containing enhanced cell death rates in both cell lines. For instance, the IC50 of B16F10 and LMM was around 25 μg/mL and 15 μg/mL CM in Figure 2A,B. However, at the higher CM concentration, such as 500 μg/mL and 1000 μg/mL, the cell viabilities in both types of melanomas were at ground levels, roughly 30% and the cell death rates reduced insignificantly at 1 μg/mL CM. Thus, 1 μg/mL, 500 μg/mL, and 1000 μg/mL CM would be excluded, and 5 μg/mL, 25 μg/mL, and 125 μg/mL CM would be conducted in the following experiments because the proliferative viability of B16F10 and LMM decreased recognizably between 5 and 125 μg/mL CM. The results distinguished that B16F10 cells were more sensitive to CM than LMM and approved the practical bio function of CM as a dosage-related cell growth inhibitor in B16F10 and LMM.

### 3.3. CM Causes Apoptosis in B16F10 and LMM Cells

Many anti-cancer mechanisms are related to apoptosis, including the caspase pathways and Bax/BCL2 proteins. Previous experiments in this study confirmed that CM could diminish the cell viability in B16F10 and LMM, and the reason was assumed as apoptosis. Therefore, the mRNA and protein of cleaved caspase-9, Bax, and BCL2 were detected in Figure 2. The caspases were a group of intracellular proteases responsible for helping the formation of apoptotic bodies during apoptosis, and the cleaved caspase-9s were situated at critical junctions in the apoptosis pathway. When cytochrome c from mitochondria was released, it triggered the activation of cleaved caspase-9s. B-cell lymphoma protein 2 (BCL2) and B-cell lymphoma protein 2-associated X (Bax) were two proteins related to the cytochrome c release. BCL2 could prevent Bax from releasing cytochrome c and restrict apoptosis downstream progress, resulting in cell survival. Some studies confirmed that a high Bax/BCL2 ratio favors apoptosis.

Figure 2C,D demonstrate the mRNA expression rate of cleaved caspase-9, Bax, and BCL2 in B16F10 and LMM individually. Figure 2E,F show the Western blots of protein quantity in cleaved caspase-9, Bax, and BCL2 in B16F10 and LMM and the protein accumulation rate illustrated in Figure 2G,H. In Figure 2C,D, the mRNA expression rate of cleaved caspase-9, Bax, and BCL2 in B16F10 and LMM enhanced because of the increasing of CM concentration. The cleaved caspase-9 and Bax were activated sharply by CM in both cell strings, but the BCL2 had only a mild increase. Figure 2G,H revealed that the protein expression in both cell strains demonstrated the same tendency, and the Bax/BCL2 protein ratio escalated in different CM concentrations. The Bax/BCL2 ratio in the control group of B16F10 and LMM were both approximately 1.3, but the percentage in both cell lines increased over 1.5 when the 125 μg/mL CM was adopted; thus, CM could induce apoptosis in tumor cells.

### 3.4. The Intracellular ROS Was Detected by DCFDA Staining

**Figure 3:**
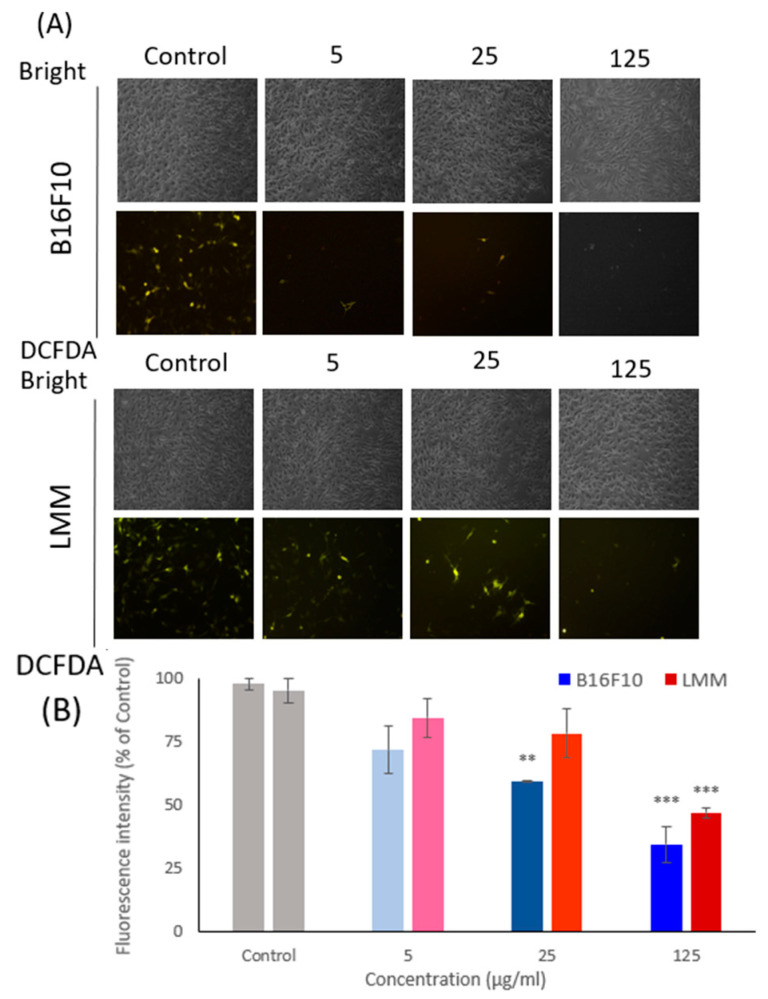
(**A**) The oxidative pressure inner B16F10 and LMM were tested by DCFDA fluorescent staining under a fluorescent microscope after conducted 5, 25, and 125 μg/mL CM. (**B**) The fluorescence intensity was analyzed in B16F10 and LMM after adopted 5, 25, and 125 μg/mL CM. ** *p* < 0.01, *** *p* < 0.005.

Intracellular oxidant stress was considered an essential motivation in inducing carcinogenesis. However, the relationship between cancer and ROS is complex. Some studies proved that the apoptosis process in cancer cells could result from extreme ROS levels, which means the superabundant or insubstantial level of ROS could lead to apoptosis. Figure 3 illustrates the ROS level in B16F10 and LMM by DCFDA staining. Figure 3A displays the cellular morphological photos of B16F10 and LMM, and the analyzed results are presented in Figure 3B. DCFDA can evaluate the intracellular ROS levels by the disclosed fluorescent brightness, and the more ROS represented, the greater fluorescence produced. Figure 3B shows that the ROS level decreased significantly because of the CM density increase. Compared to the effects of CM toward B16F10 and LMM, B16F10 is more sensitive to CM existence, and the ROS level had a significant drop in the experiments. In contrast, the LMM had more resistance toward CM.

### 3.5. CM Inhibits LMM Migration

**Figure 4:**
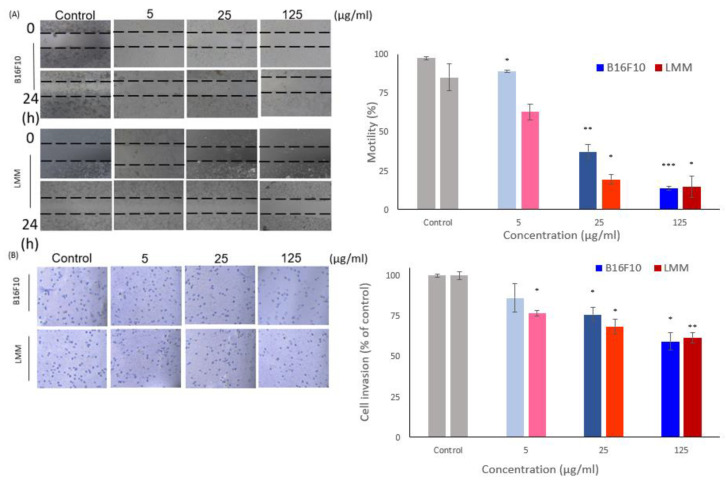
(**A**) The horizontal migration ability of B16F10 and LMM were tested by wound healing assay after co-incubating in different concentrations of CM. The results are shown in a microscope photo and analyzed in a histogram. (**B**) The vertical invasion ability of B16F10 and LMM was examined by Boyden invasion assay. The results are revealed in microscope photos and analyzed in a histogram. * *p* < 0.05, ** *p* < 0.01, *** *p* < 0.005.

The main reason for cancer-associated death is cancer metastasis. Cancer cells can escape from the primary tumors and invade neighboring tissues as metastases because of the deregulation of cell migration. This research used the cell wound healing test and the Boyden invasion assay to investigate horizontal and vertical aggression rates. Figure 4A showed B16F10 and LMM in the microscopical photo of horizontal cell migration from the beginning and 24 h after being treated by different concentrations of CM and analyzed the results. In control, 5, 10, and 15 μg/mL CM, B16F10 mobility rates were 67.3%, 21.9%, and 12.56% and the cell mobility rates in LMM were 62.8%, 19.3%, and 14.5%. The experiment showed CM could reduce the horizontal migration in B16F10 and LMM, and the inhibiting rates were proportional to the CM concentrations. There were insignificant differences in the horizontal cell migration rate between B16F10 and LMM.

Figure 4B shows the results of the Boyden invasion assay, which is evidence that CM affected the vertical invaded rate in B16F10 and LMM. Different thicknesses of CM treated the cells on the above plate, and they would invade the blow plate through the semi-permeable membrane. Then, the cell quantity on the lower plate could be counted, representing the vertical invasion level of tumors. CM increased the concentration while the vertical aggressive rate in B16F10 and LMM regulated downward, around 25% of migration rate was restrained in 125 μg/mL CM. Figure 4B confirms that CM could prevent cell metastasis effectively in both B16F10 and LMM in the horizontal and vertical directions. However, CM had better inhibition ability in the horizontal migration than in vertical movement in B16F10 and LMM.

### 3.6. CM Increased the Efficacy of CD8+ T Cells

**Figure 5:**
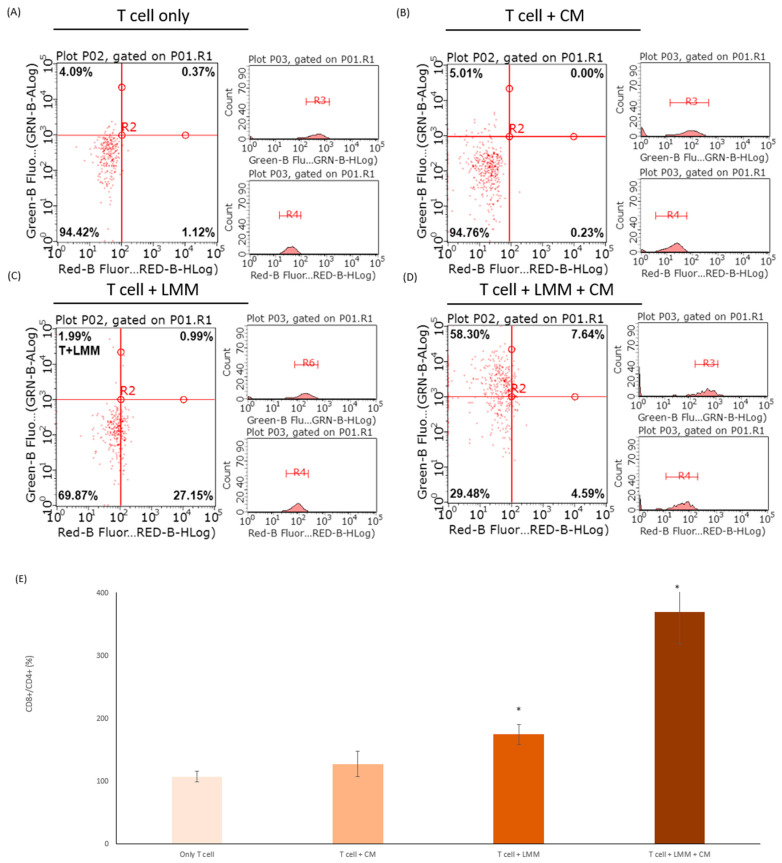
The cells with CD8+ and CD4+ cell markers were measured in (**A**) T cells, (**B**) T cells incubated with CM, (**C**) T cells cultured with LMM, and (**D**) T cells co-incubated with LMM and CM by flow cytometry. (**E**) The histogram of the ratio of CD8+/CD4+ in the four experiment groups above. * *p* < 0.05.

It was essential to generate durable memory CD8+ T cells in the tumor microenvironment for curing cancer. However, producing and regulating a functional CD8+ T cell remained unknown [15]. This study revealed the relationship between CM, melanoma, and T cell. The CD8+ and CD4+ cell markers were double-stained and divided the experiment groups into four groups (Figure 5A). After 24 h, the ratio of CD8+/CD4+ was analyzed and measured by a flow cytometer. The CD8+ and CD4+ were the markers of T cells, which CD8+ and CD4+ cell markers could distinguish the difference between the cytotoxic T cell (CD8+) and other types of T cell (CD4+). Therefore, the CD8+/CD4+ could represent the related quantity of activated cytotoxic T cells. The T cells only cultured with CM had a slightly higher CD8+/CD4+ ratio than the control group, but there was no significant difference between those two groups (Figure 5B). In Figure 5C, T cells were incubated with LMM, the number of cytotoxic T cells increased as twice the control group, which meant LMM could activate the cytotoxic T cell. The last group included T cell, LMM, and CM into the same plate, and the CD8+ ratio was sharply boosted, which was almost four times the control group (Figure 5D). Those experiments confirmed the CM function in increasing the T cell activities.

## 4. Discussion

Free radicals in the body are substances produced by oxygen metabolism and react exceptionally with any armamentarium. This means ROS in cells needed to stay at a dynamic level to proliferate cells and the superabundant and meagerly level of ROS could promote cell apoptosis either way [19]. When the free radical levels in the human body exceed the normal levels, the chain reactions that may facilitate the oxidation of essential substances could be triggered to eliminate the ROS into the average level. The healthy human body contains chemicals protecting from free radicals and reduce possible harm. These chemicals can reduce the rate of cell division and repair the damage caused by free radicals in many cases [20]. Furthermore, tumor progression and metastasis result from an overload of free radicals in the tumor microenvironment. Therefore, justifying the level of free radicals could be a great strategy to cure cancer.

In this study, CM showed an excellent capability for removing ROS, different capacities in reducing power, ferrous ion chelating, and DPPH free radical scavenging test and was influential in eliminating intracellular ROS. In the experiment, CM could reduce the inner cell ROS in both B16F10 and LMM even though LMM had more resistance than B16F10 in the lower level of free radical. The ROS levels sharply declined in cancer cells provoked the mRNA and protein level of cleaved caspase-9 and Bax. In contrast, the mRNA and protein level of BCL2 increased slightly. It resulted in the cleaved caspase-9 and Bax/BCL2 being higher than the regular quantity, which reduced the cell viability of B16F10 and LMM. CM could trigger cell apoptosis in melanoma, which was proven in this experiment.

Melanoma is one of the most aggressive tumors and exposure to ultraviolet (UV) radiation is one of the main causes of skin cancer. Some studies show that the spread of melanoma is rapid and systemic treatments for melanoma and metastatic melanoma are usually ineffective. Those properties of melanoma develop into the main reason for death in skin cancer. In clinical studies, the development of various tumors was linked to overloaded oxidative stress, which can directly cause DNA damage [17,21]. More-than-sufficient ROS in the tumor microenvironment could stimulate resistance to cancer-curing drugs. Melanoma also showed immune escaping and migration ability enhancement when the tumor microenvironment had a higher level of ROS. This study showed CM could reduce the speed of horizontal and vertical invasion in B16F10 and LMM effectively and had a dose-dependent trend.

Apoptosis is one of the most effective defense methods in the immune system, and the lack or excessive accumulation of lymphocytes causes profound consequences [22]. Autoimmunity is a condition in which the repose of immunocytes acts out of control. T cells are in the center of the apoptosis process in the human immune system, especially against the generation of cancer cells. However, excessive or premature apoptosis can lead to the development of cancer [23]. Thus, the regulation of apoptosis by regulating T cells could be an effective strategy for cancer treatment. The immune cells themselves will not change from CD4+ to CD8+. If there are a lot of changes after adding the drug, it should be verified whether there will be immune solid side effects after adding the compound. We know that adding the component itself will not promote a large amount of CD4+ converting to CD8+. We suspect that the addition of cancer cells will inhibit immune cells from transforming into killer T cells, and the addition of pharmaceuticals will help immune cells to identify cancer cells and transform into killer T cells to attack cancer cells [24,25]. The effects of CM in promoting T cells and apoptosis have been explored in these experiments and showed a significant result. When T cells were incubating with LMM, the CD8+ T cells were more active with CM conducted into the medium. The outcome may be giving new therapy options in treating carcinoma in situ and metastatic melanoma.

## 5. Conclusions

**Figure 6:**
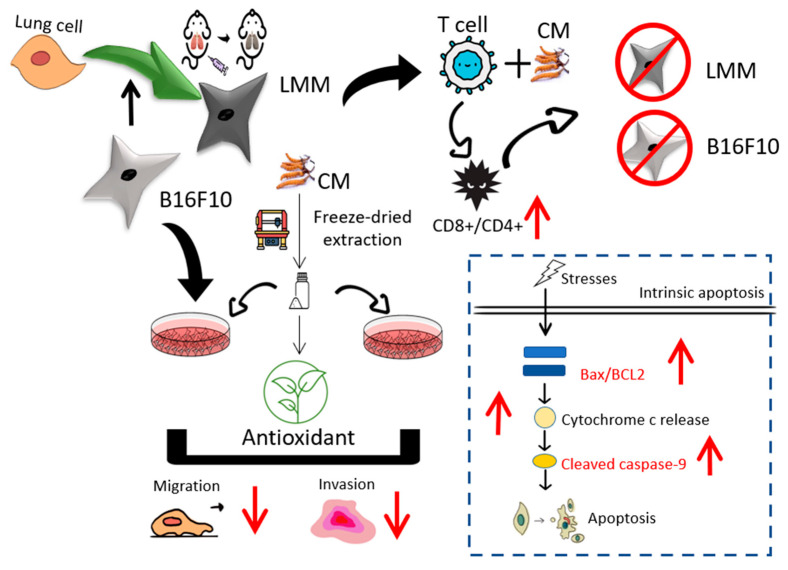
The process and results of this study.

Many studies proved that CM benefits health in many ways. In this study, three important conclusions have been confirmed, in which CM had antioxidant, anti-carcinogenic, and CD8+ T cell activation properties. First, CM had a significant ability in dismissing ROS by reducing power, ferrous ion chelating, and DPPH elimination, and CM could also decrease the intracellular ROS level. Second, CM could be the cancer treatment by enhancing the apoptosis progress, depressing tumor cell viability, and suppressing the migration rate of melanoma. CM could shrink the horizontal invasion and vertical migration abilities in B16F10 and LMM. It could decrease the cell viability of B16F10 and LMM by activating the apoptosis program, and this study confirmed the increase of the mRNA and protein level of cleaved caspase-9 and Bax/BCL2 in both cells. Finally, adding cancer cells will inhibit immune cells from transforming into killer T cells, and the addition of CM will help immune cells to identify cancer cells and transform them into killer T cells to attack cancer cells. The ratio of CD8+/CD4+ in T cells, which CM adopted, increased significantly (Figure 6). Those three pieces of evidence indicate that CM could inhibit and treat melanoma.

## Figures and Tables

**Table 1:** Reducing power, ferrous ion chelating, and DPPH were used to test the ROS removing ability in CM.

Concentration (µg/mL)	Antioxidant Capacity		
	Reducing Power (Absorbance at 700 nm)	Ferrous Ion Chelating (%)	DPPH (%)
Positive control ^a,b,c^	0.77 ± 0.16	96.86 ± 7.90	89.43 ± 0.65
1	0.23 ± 0.14	18.95 ± 2.13	12.53 ± 1.78
5	0.25 ± 0.08	26.26 ± 3.06	20.31 ± 0.24
25	0.29 ± 0.09	36.53 ± 3.61	31.73 ± 2.09
125	0.32 ± 0.16	52.99 ± 1.85	40.72 ± 3.67
500	0.54 ± 0.06	69.12 ± 2.59	50.87 ± 9.44
1000	0.60 ± 0.07	77.78 ± 6.80	69.70 ± 2.75

^a^ BHA was used as a positive control on reducing power at 100 mM. ^b^ EDTA was used as a positive control on ferrous ion chelating ability. ^c^ Vitamin C was used as a positive control on the DPPH assay.

## Data Availability

Data are contained within the article materials.
